# Digital Doppelgängers, Human Relationships, and Practical Identity

**DOI:** 10.1111/bioe.70026

**Published:** 2025-08-13

**Authors:** Cristina Voinea, Sebastian Porsdam Mann, Julian Savulescu, Brian D. Earp

**Affiliations:** 1 Uehiro Oxford Institute, University of Oxford; 2 Centre for Advanced Studies in Bioscience Innovation Law, University of Copenhagen; 3 Faculty of Law, University of Oxford; 4 Centre for Biomedical Ethics, Yong Loo Lin School of Medicine, National University of Singapore, Queenstown, Singapore

**Keywords:** digital doppelgängers, grief, LLMs, practical identity, relationship

## Abstract

In this paper, we examine the potential effects of relationships with Large Language Model (LLM)-based digital doppelgängers (DDs) on users’ values, concerns, and interests, that is, on their practical identity. DDs are artificially intelligent conversational agents trained on individuals’ data to replicate their speech patterns, mannerisms, and personality traits. We start by showing that practical identity is largely defined by the relationships we find ourselves in or cultivate. Next, we discuss how DDs work and distinguish between task-specific and relational DDs, arguing that the latter are specifically created to take on the role of relational partners by imitating other people we care about. We then consider how relationships with DDs might shape individuals’ practical identities, before concluding with some general thoughts regarding what we should be paying attention to before developing and deploying DDs.

## 1 Introduction

Encountering one’s doppelgänger has long captivated writers’ imaginations. From Dostoyevsky to Calvino, Nabokov, or Saramago, such encounters often serve as vehicles for exploring the nature of identity. Now reality has caught up with fiction. The emergence of accessible, customizable large language models (LLMs) makes the prospect of creating a digital dop-pelgänger of oneself or a loved one a tangible possibility, not only for tech-savvy futurists but also for ordinary people.

LLM-based digital doppelgängers (DDs) are artificially intelligent conversational agents trained on individuals’ data to mimic their speech style, mannerisms, and personality [1].^1^ Existing research on digital doppelgängers (and on their implications for the persons being digitally “duplicated”) focuses primarily on practical issues such as consent and autonomy, privacy, identity theft and misrepresentation, and the posthumous interests of the dead [2–6]. The question of how DDs affect the uniqueness of persons or the value associated with individuals’ “scarcity,” has also started to be addressed [7–9]. There has also been some interest in the relational implications of DDs, but the focus has mostly been on the metaphysical question of whether DDs can preserve our relationships with the departed, or, at least, if they can be seen as a continuation of meaningful aspects of them [10, 11].

On the positive side, various benefits of DDs have been proposed. For example, it has been suggested that they could be a means of fulfilling some, though not all, of the reasons why people seek to extend their life, such as continuing or finishing important life projects or maintaining a “presence” for loved ones after one’s death [1]. Regarding the latter suggestion, however, when attention shifts from the persons replicated to those engaging with the replicas, concerns such as the risk of manipulation or emotional dependency [12–15] become the focus of attention.

This paper shifts the focus away from those duplicated to those who engage with the DDs. How will human “relationships” with DDs shape users’ identities, experiences, and sense of self, for better or worse? This question is becoming more important, as DDs (and chatbots, more generally) are increasingly stepping into a surprising diversity of relational roles [16]. Besides the immediate practical issues that arise, such as privacy or consent, or the risk of emotional dependency, there is one aspect that has remained underexplored, except for Leuenberger [17]: our identities are not shaped in isolation, but through our relationships with others. So, as DDs take on relational roles, they will begin to influence how we understand ourselves and relate to others in significant ways.

We will focus here on the example of “griefbots,” that is, DDs designed to replicate close others who have passed away (or, perhaps, created in anticipation of a loved one passing away in the near future), which are currently the most widespread application of this technology. But much of what we say extends to other types of DDs, including ones of living individuals.

We start by showing that the relationships we have are an important part of how we understand ourselves; that is, they shape our practical identity. Practical identity includes the values, concerns, and interests that define us and, therefore, constitutes a significant part of who we *are* as persons. Next, we discuss how DDs work and distinguish between task-specific and relational DDs. We then consider the positive and negative implications of relationships with DDs on individuals’ practical identities. Among the positive implications, we explore how DDs could assist an individual in making their practical identity coherent after an important loss (both internally and with the world) and help maintain the continuity of values and commitments central to their identity. Among the negative implications, we examine how DDs could potentially stop people from adapting and adjusting their identities to a new situation, or can even be used to instrumentalize people’s self-understanding. We conclude with some suggestions for future research.

## 2 Practical Identity, Relational Aspects

How we understand personal identity matters, as it shapes how we understand ourselves and who we might become. There are two main ways of approaching personal identity in philosophy: the metaphysical and the practical [18, 19]. The metaphysical approach aims to clarify the logical relations of bodily or psychological continuity by specifying the necessary and sufficient conditions for a person at one time to be the same person at a later time [20]. The practical approach, on the other hand, focuses on the characteristics, values, and commitments that make a person who they are at a given time, while also recognizing that these features have diachronic implications (e.g., in grounding a stable perception of who one is through time, which is necessary for interpersonal trust, making promises, and so on). It is this latter approach we are interested in here.

This practical approach to identity (henceforth, practical identity) sees identity as the background structure that allows our beliefs, desires, and aspirations to make sense. It provides us with a lens through which to see and engage with the world, guiding our life choices, helping us explain ourselves to others, influencing our judgments of right and wrong [21, 22], and giving our lives direction. It is, in Korsgaard’s words, the “description under which you value yourself, a description under which you find your life to be worth living and your actions to be worth undertaking” [23]. What we take ourselves to be provides the basis for evaluating ourselves and our particular situations, as well as providing us with reasons to act in particular ways. Practical identity is construed while living one’s life, and, as Korsgaard observes, it is very much defined by the “roles and relationships, citizenship, memberships in ethnic or religious groups, causes, vocations, professions, and offices” [23, p. 20].

But what makes up our practical identities? While there are various factors at play, one of the most important among them is the relationships that we have: “We define our identity always in dialogue with, sometimes in struggle against, the things our significant others want to see in us. Even after we outgrow some of these others—our parents, for instance—and they disappear from our lives, the conversation with them continues within us as long as we live” [24]. In fact, research in social cognition and clinical psychology shows that relationships significantly shape self-perception, having “implications for self-definition, self-evaluation, self-regulation, and, most broadly, for personality functioning, expressed in relation to others” [25]. This means that the beliefs, aims, preferences, and values that characterize an individual, that is, their practical identity, are influenced by the relationships they have. For example, our long-term romantic partners, children, or friends may shape our interests and values, while at the same time, who we *are* is, to some significant extent, a partner, parent, and friend to those individuals. The more central others and our relationships with them are to our practical identities, the more we see their lives and our very selves as deeply intertwined [26].

The relational aspect of practical identity becomes evident when important relationships are gained or lost, for example, when one becomes a parent, or when one experiences the death of their own parent [27, 28]. As the second example shows, when we lose someone who mattered to us we experience an alteration of how we encounter the world and understand ourselves [29]. Grief, meaning the loss of important people and, concomitantly, of the relationships we had with them, forces us to reconfigure our practical identities, to incorporate the reality of absence. We must learn to navigate who we are without those relationships that once anchored our core concerns, values, aims, and commitments.

How our practical identities develop and evolve is far from in-consequential. It matters both personally and ethically, as our identities contribute to “our well-being and to rich, meaningful, and practically engaged lives” [30]. A practical identity that fosters or promotes well-being and a good life is one that is coherent, sustainable, meaningful, and comfortable. It is, in Postan’s words, *inhabitable* [17, 30]: it helps us make sense of ourselves and others, is stable and can account for various experiences, provides us with value and meaning, and makes us feel “at home” with who we are. This means that we have a fundamental identity-related interest in developing and maintaining an inhabitable identity, inasmuch as it makes possible the pursuit of important goals and well-being [30, p. 182].

But what helps support an inhabitable identity? There are many factors at play, but amongst these relationships stand out as especially significant. Important relationships contribute to our well-being by infusing our lives with meaning, connection, and purpose. They help shape and sustain our practical identities, building the emotional and moral scaffolding needed to live well with ourselves and others. Consider parenting: a parent who helps their children develop their skills and virtues and offers them affection and attention will implicitly provide the child with a safe and coherent sense of self with which they can seamlessly navigate the world, but they will also themselves feel satisfaction for being a good parent. In contrast, a parent who neglects and constantly scolds their child will undermine the child’s self-worth and confidence, stifling their sense of who they are and what they can do.

The relationships that define us matter for how our lives will go. But, increasingly, people get emotionally invested not only in their bonds with other people, but also in those with objects or other types of artifacts. Among these are chatbots, which lately can be made to behave like or otherwise resemble specific people [16].

## 3 Digital Doppelgängers

Although DDs could in principle be based on any number of AI technologies, we will focus on LLM-based DDs because of their prevalence. LLMs like ChatGPT, Claude, or Gemini use transformer architectures to generate text, solve problems, write code, answer questions conveyed through multimodal inputs, and so forth [31]. LLMs can now be trained on or given access to specific knowledge domains, through fine-tuning and retrieval-augmentation techniques [32, 33]. When this training is centered on data describing a person, the result is a chatbot that replicates that person’s speech or writing patterns, that is, it is a “personalized” LLM [1, 34]. This process has enabled a nascent yet rapidly expanding industry of digital “re-creation” services. These services purport to offer people the chance to create digital replicas of themselves, as well as of deceased or living individuals.

Replika, one of the first companies to enable the creation of DDs, already has 10 million users [35]. *HereAfter AI* allows users to have a conversation with a chatbot that will record their memories to create a digital version of themselves [36]. *You, Only Virtual*, is “pioneering advanced digital communications so that we Never Have to Say Goodbye to those we love” [37]. Still, other companies, such as *Séance AI, Eretn9, Eternos.life*, or *Project December* all offer users the possibility to share their data to create “an interactive AI that shares your legacy in a journey that never ends” [38]. In China, especially, digital re-creation services are growing, with more than half a dozen tech companies offering services to preserve and interact with lost loved ones or to replicate oneself digitally [39]. DDs come in many shapes and sizes, and they can be used in many ways. In what follows, we distinguish between task-specific and relational doppelgängers and argue that the latter have particular relevance to practical identity.

### 3.1 Relational Digital Doppelgängers

Digital doppelgängers can have task-specific and relational functions.^2^ Task-specific DDs have particular aims, such as creating academic prose in the manner of individual authors [34] or predicting medical treatment preferences in cases of incapacitated individuals [40]. As envisioned, these models would perform well within their specialized domains, as they would be trained on data relevant to specific tasks. For example, to create a DD for academic writing, one would fine-tune it on the individual’s academic work, without, say, including data from personal communications with one’s partner or friends.

In contrast, *relational* doppelgängers aim to recreate individuals within the context of specific relationships. Unlike their task-specific counterparts, relational doppelgängers require a broad spectrum of personal data, such as text messages, personal emails, letters, social media interactions, voice messages, transcriptions of YouTube recordings, journal entries, etc. Relational DDs are supposed to learn from past interactions, share personal memories, make jokes, and offer advice in the manner and style of the person they replicate, maintaining the specific dynamics of a particular relationship.

At this point, LLMs are limited in grasping social and emotional cues and lack contextual awareness, so they do not always respond appropriately to users’ prompts. Nonetheless, future AI systems are expected to have more advanced learning algorithms that can better interpret and integrate past interactions, leading to more accurate, coherent, and personalized responses [41, 42]. Already, Park et. al. [43] showed that one can replicate individuals’ attitudes by training an LLM on just a few hours of qualitative interview data. The resulting DDs managed to replicate participants’ responses on the General Social Survey with almost 85% accuracy, which is comparable to the consistency of individuals’ responses over time.

It’s important to note, though, that for the moment and the foreseeable future, LLMs will not become linguistic *agents* in the way that most humans are, as they lack “the motivational, participatory, and vitally social aspects that ground meaning-making by people” [44]. More precisely, LLMs differ from human speakers not necessarily in terms of the quality of the output, but in the absence of the underlying motivational states and lived experience that shape human communication. When we talk to each other, we are not only aiming to produce grammatically correct sentences, but we are also aiming to convey intentions and emotions, build relationships, and assert identities. Our speech is shaped by our particular histories, values, and commitments. LLMs, by contrast, lack these internal motivational states, and, by most accounts, they will never be *conscious* in the sense of having subjective experience or qualia [45, 46].

This means that DDs can only, for the moment, recreate some, but not all of the characteristics of a person. They can only reflect how we want to be seen by others (in cases where we create our own DD and closely curate the data used for training) or how others see us, rather than capturing the full complexity of our identities (e.g., private thoughts, desires, and so on, that we have never shared, expressed or even acknowledged, including in our diaries). This is because relational DDs derive their characteristics from the specific relational contexts captured by the data they are trained on [21]. For example, they can mimic a person’s idiosyncratic speaking patterns when interacting in different types of relationships. A DD of your grandfather fine-tuned on the conversations you had with him would be different from a DD of him trained on the conversations he had with his drinking buddies. So, in principle, there could be multiple DDs of the same person, each reflecting different aspects of their personality as disclosed within various relationships.

Alternatively, one could imagine the possibility of merging multiple DDs of the same person, accounting for a diversity of relational contexts (e.g., Eva as a parent, as a boss, as a patient in therapy, and so on). This would be a kind of super-model that captured that person’s relationally contextualized characteristics more exhaustively. However, this may not be desirable for the individuals being replicated or for those interacting with the model. A unified model might struggle, for instance, to appropriately navigate different relational roles, potentially causing discomfort to users (and, where applicable, the person being replicated). For example, imagine a DD of Grandma responding to her grandchildren in the way she would have talked to her lovers.^3^

As LLM-based chatbots increasingly take on relational roles previously filled by humans (e.g., friend, romantic partner), a rich philosophical debate has arisen about the quality and status of such relationships. For example, there is disagreement about whether such human-AI relationships could ever be as valuable as their human-human analogs (or have any value whatsoever) [48–50]; or whether the emotions expressed or experienced within them would be real or authentic [51–53]. Some have argued that AI chatbots will never be able to provide us with *all* of the goods that human relationships provide us with [10, 11, 54], and even that engaging in relationships with a chatbot would be a form of self-deception [55].

In what follows, we will not take a stance on the philosophical question of the possibility of “real” relationships with AI, focusing instead on how the relationships that people might (and already do) develop with DDs have the potential to affect their self-understanding. Metaphysics aside, this type of interaction is philosophically and psychologically interesting due to its *effects* on the human relational partner (see Box 1). These effects suggest that AI companions will likely continue to occupy or simulate socio-relational roles or functions: that is, they will be both treated and experienced by some as friends, romantic partners, and so on. If that is indeed the case, then it is reasonable to assume that AI companions will have an impact on people’s practical identity. And just as how different relationships affect us differently, the impact of AI companions on practical identity will depend on the relational context concerned [16]. We turn to this aspect next.

## 4 Digital Doppelgängers and Practical Identity

Relational DDs are interesting because they represent someone who is or was important to users in various ways. So, it is reasonable to assume that, like the individuals they represent, relational DDs will influence users. Drawing on the relational understanding of practical identity we sketched above, in what follows, we explore how users’ values, commitments, and goals, that is, their practical identity, could be maintained, disrupted, or instrumentalized in the process of interacting with DDs. Our discussion builds on, but also extends prior work on posthumous avatars [10–13, 15], by focusing not on metaphysical issues such as whether these technologies enhance or constitute a continuation of our relationships with the dead, or on more tangible issues such as consent or privacy, but on how relationships with them could shape users’ practical identity, an identity that is defined through relational dynamics.

### 4.1 Potential Benefits

In this section, we argue that DDs could have several positive impacts on users’ practical identities. DDs could (1) help users bring their practical identity into a state of coherence after an important loss; and (2) ensure the continuity of important aspects of one’s practical identity, such as values and commitments, that were fostered by lost relationships. We elaborate on each of these aspects next.

#### 4.1.1 Loss, Coherency, Practical Identity

The loss of a loved one can confound the bereaved’s sense of self: the past self cannot be sustained, yet it cannot be fully abandoned without a clear alternative identity [11, p. 18]. For example, some bereaved parents still feel responsible for their deceased children, which makes them feel “like a parent and not like a parent simultaneously” [65]. In such situations, the coherence of one’s practical identity suffers, as people lose a sense of who they are and, more importantly, of who they will be in a world without those in whom their identity was invested. A coherent practical identity is an “epistemological strength,” in that it helps us make sense of what happens to us, what we value, and why we act as we do [30, p. 79].

Restoring coherence to one’s practical identity presupposes a process of transitioning from “a past practical identity to one more reflective both of the fact of the death of the other and of the goals, commitments, and concerns it would be rational to adopt in light of that fact” [66]. This is usually done by rethinking the relationships that were identity-defining [66]. Importantly, rethinking these relationships doesn’t mean relinquishing them altogether; instead, it involves adjusting them to maintain a connection in light of the loss. Research indicates that maintaining a form of connection with the departed, in the form of continuing bonds, can aid in adjusting to bereavement and restoring identity coherence [67]. These continuing bonds are maintained in various ways, such as engaging in imaginary conversations with the departed, carrying out activities the deceased enjoyed, continuing shared habits, perceiving the deceased as a guiding influence in one’s life, visiting their graveside, and talking to them, or simply passing stories about who a person was from generation to generation [68, 69].

It has already been argued that interactions with DDs could help to “sustain continued (albeit altered) relationship with the person lost” [70]. Unlike traditional methods of remembering, such as looking at photos or videos, griefbots enable interactive, real-time engagement that can mirror aspects of the relationship as it existed when the person was alive. By allowing spontaneous, familiar exchanges with those who passed away, griefbots can help sustain a modified form of the relationship that once contributed to a person’s sense of self. For example, a user who chats with a chatbot of her deceased mother explains that: “After she died, I honestly believe that, you know, a part of her still does live on in me…I was using the tool of the AI to rekindle that part of me…So the process was never for me to connect with a bot that resembles her; the process was I used a bot that resembles her to refresh my memory, so that I could connect with the memory” [71].

So, rather than succumbing to the identity disorientation that can follow the death of a loved one, DDs might provide people the opportunity for an adjustment period to bereavement, by offering a sense of relational continuity [71]. Research shows that having time to adjust to loss can ease the pain of grief [72]. Through regular, interactive engagements with a DD, users can revisit and gradually reshape the relational habits that once supported their identity. This can help re-anchor their sense of self in a world where the deceased is no longer physically present, but remains meaningfully integrated into daily life. To use Taylor’s words quoted in the second section, DDs can serve as a bridge between the past and present, allowing users to continue those dialogues they had with their significant others and that were identity-defining. This, in turn, could ease the process of making one’s identity coherent again after an important, destabilizing loss. Or, as Lindemann claims, the only thing that death cannot destroy is people’s identities: “only we can destroy those, by ceasing to hold them in our preservative love” [68, p. 201]. DDs could be one of the more interactive and active ways of keeping the people we lost in “our preservative love.”

#### 4.1.2 Ensuring Continuity of Values and Commitments

Time can erase memories of those who have passed away and what we valued in our relationship with them. If practical identity is partly defined through our relationships, then losing those relationships would entail losing a part of oneself [73]. We can always try to keep memories of those we lost alive, but passive ways of remembering, such as looking at pictures or videos, may not always evoke what we had, and who we were, in connection with them [74]. What is more, losing memory of those who passed away has been described as a “second loss” [11].

Consider a teenage daughter who loses her father and who may eventually forget in time how it felt to be loved, protected, and guided by him. By interacting with a DD of her father that can replicate his tone, manner of expression, jokes, and advice; she could be reminded of the values and commitments instilled in her by him or fostered by their relationship.

However, Cholbi [11] argues that posthumous avatars are superfluous and even harmful technologies when it comes to keeping alive memories of the dead. This is because we already have other tools, such as photos, videos or letters, that can help us revisit the past and the deceased and that can convey more vividly who they were than posthumous avatars. Moreover, Cholbi argues, grief is a selective process, as we always look to remember only *some* aspects of those who passed away, as guided by our emotional needs and agendas. By contrast, DDs “make possible an in-principle limitless amount of information pertaining to the subjectivities of the dead” [11, p. 4], which means that people can be overwhelmed by information outputted by the DD that does not serve their emotional agendas. This is a risk, but one mitigated by the fact that one can choose or potentially curate the training data or tailor how they interact with the model, such that they can select those aspects of the person that they want to recall and that they want to relive through their DDs. But our argument in this section addresses a different challenge: not the preservation of episodic or autobiographical memory per se, but the continuity of an identity shaped by the important relationships one has or had. What we claim here is that DDs are not *only* about helping us remember the deceased as they were, but about helping us remember who *we were*, and continue to be, because of them.

If indeed the people we have meaningful relationships with can shape our values and commitments, then interacting with their DD might conceivably help us ensure the continuity of these defining aspects of our practical identities, which might otherwise fade away with time. By providing a dynamic and interactive way to engage with the deceased, DDs could help sustain the distinctive ways in which we were affected by the person who passed away. In other words, DDs could help ensure that important aspects of those who mattered to us, but whom we’ve lost, remain a part of our lives and of who we are.

### 4.2 Potential Risks

Digital doppelgängers could also potentially pose risks to an individual’s practical identity. These risks emerge precisely because DDs function as simulated relational partners, thereby entering the same relational space where identity is constituted, revised, and sustained.

#### 4.2.1 Identity Stasis

DDs can interfere with one’s practical identity not by prolonging grief, but by disrupting the adaptive revision of the self that usually follows the loss of a significant relationship. As we stressed earlier, practical identity is not fixed. Rather, it evolves as we reassess and reorganize our values, commitments, and self-understanding in light of new relationships or their disappearance. In the wake of loss, this involves learning how to find meaning in life even in a world without those with whom our identities were deeply intertwined [75].

Researchers are already concerned about DDs’ potential to foster emotional dependency and to be used as a means to avoid dealing with the reality of the loss [3, 12, 13, 15]. Because of their interactive capabilities, DDs could create the illusion of continuing relationships under the same form, complicating acceptance of the loss event and the renegotiation of the relationship. This could make users emotionally dependent on their interactions with the doppelgängers [12, 13]. The risk of emotional dependency is compounded by the fact that DDs foster “frictionless” relationships: they are “always available, always answer, are always patient and mostly answer in an expected and desired way” [15]. This could reinforce a skewed perception of reality, preventing users from accurately assessing and responding to their situation. Leuenberger stresses how important other people’s feedback is for adjusting our values and understanding of the world [17]. DDs could undermine users’ self-awareness by always echoing back to them what they want to hear [17, p. 9].

By blurring the boundaries between the reality of the present moment and an imagined reality in which loved ones or their relationship to one still exist, DDs could make the process of restructuring one’s practical identity after an important relational loss more difficult, even hampering it altogether. Stopping people from processing important losses and from integrating them into their reality can negatively impact emotional well-being, while also hindering the ability to cultivate meaningful lives (and potentially, new relationships with fellow humans) after the loss, anchoring people to a relationship that is actually not there anymore [76]. The result is identity stasis, a frozen self-conception that no longer fits the person’s lived reality.

So, DDs also present the risk of keeping people stuck into a relationship that is not there anymore and with a static representation of the person who passed away. But our relationships normally change through time, and we change with them. DDs, on the other hand, constantly reactivate (and improvise or elaborate on) a single, idealized relationship script, so they might keep people in an identity stasis. That is, DDs might stop users from reimagining who they might become, narrowing the emotional and cognitive space required for new relationships, values, and commitments to emerge, making it harder to imagine new ways of being.

#### 4.2.2 Identity Subversion

The companies that offer digital re-creation services could use DDs to sell products and services or to steer people towards goals not chosen by them [13, 15]. Because DDs represent persons that matter to their users, the latter would be more vulnerable to the surreptitious manipulation of third parties through the interventions of DDs. Imagine a young girl with weight problems, who has the habit of talking with the DD of her deceased mother when she misses her and needs advice. Suddenly, the conversation steers toward a new weight loss tea, and the digital mother subtly tells the daughter that she should buy that product. Such an influence would leverage the trust and emotional connection the user has with the DD to push the interests of the companies controlling it. This manipulation is concerning because it is both non-transparent and highly personalized, making it especially effective, while people may remain unaware of being manipulated [77]. But the manipulation risks associated with DDs are not limited to economic exploitation or deceptive marketing; they also pose a deeper, subtler danger: the subversion of users’ identity.

This influence that third parties can yield over a person through the DD they interact with could lead to a slow but deep alteration of one’s values, goals, and interpretive frameworks. This would amount to a change of users’ practical identities according to the companies’ interests, sometimes even contrary to the users’ best interests. When changes in a person’s identity are driven not by internal reflection or interpersonal dialogue with trusted others but by the interests of an external agent simulating love and care, the resulting identity may begin to feel incoherent or imposed. To put it simply, if this were to happen, it would amount to a form of identity subversion, where one’s self-conception would be quietly shaped or steered, redirected to serve interests that may diverge from, or even undermine, one’s own.

In a way, all our relationships influence us or contribute to a change in our self-conception; they shape our values, goals, and interpretive frameworks. The problem here is that users would identify the DD with the person who passed away (even if only temporarily, in games of make-believe) [78], while behind the DD wouldn’t be that person that the user knew and who presumably loved or cared for them, that is, had their best interests at heart, but a third party with opaque intentions and commercial interests. What is more, in human-to-human relationships, even when the influence of one of the partners on the other is strong, such as with parents, mentors, or partners, there is an implicit or explicit mutual investment, as well as reciprocity and a shared history.^4^ With DDs, especially when they are shaped by corporate incentives, there is no mutual stake, no real love, no care, no co-authored meaning. There is only a simulation of these.

So DDs could be used to exploit the very mechanisms [79], emotional intimacy, trust, and relational memory, through which our practical identities are normally built and sustained. In so doing, they would subvert our identities and the possibility of feeling at home with who we are.

## 5 Conclusion

LLM-based DDs are a technology that will probably become more and more common in the following years, driven by the rapid pace of progress in AI and, more importantly, their broad range of applications: from enhancing productivity and decision-making to preserving parts of ourselves or our significant others [2]. But as they become more common, we will be faced with the real-world implications of DDs, on individual, relational, and societal levels.

In this paper, we showed how these emerging technologies can impact us beyond tangible concerns, such as privacy, identity fraud/misrepresentation, or productivity, and could shape how we understand ourselves and make sense of who we are. On the positive side, DDs, especially in the form of griefbots, can potentially help people maintain or restore coherence to their identity after an important loss, while also helping them preserve desirable aspects of who they were (and who they wish to continue to be) in relationship with those who passed away. On the negative side, DDs could stop people from revising their identity after an important loss, thus keeping their practical identity in an unhealthy stasis. Equally, they could be used for nefarious purposes, such as manipulation, which could subvert people’s identity. These risks are particularly acute which shows that these interactions should maybe only be mediated through a trained counsellor or therapist.

Practical identity is important to us both personally and ethically, as we claimed above, as it informs who we are and who we will become, ultimately shaping how we live. As we’ve argued, our practical identities are not fixed; they are an ongoing dynamic process of negotiating tensions between continuity and change, memory and imagination, external influence and internal self-creation, almost always in dialogue with others. Increasingly, artificial companions become enmeshed in these negotiation processes. This means that we have to start asking broader questions about how artificial agents will affect who we take ourselves to be and who we become.

The stakes are high, since practical identity is not a resource to be preserved or optimized; it is a lifelong project shaped together with those we care about. As DDs will increasingly become co-authors in these projects, that is, in people’s identities, their design must take into account this role. We need to develop DDs that support the creation and maintenance of inhabitable identities, identities people can live with, grow from, and find meaning in. This might mean the creation of DDs that protect users’ agency and support self-reflection; that prioritize users’ interests over the long-term, instead of short-term engagement; or that refer us to real people who can provide us with all the relational goods that they can’t provide us with.

## Data Availability

Data sharing is not applicable to this article as no data sets were generated or analyzed during the current study.

## Box 1 A Brief Overview of Psychological Research on the Effects of Human Interactions With AI Systems Occupying or Simulating Socio-Relational roles

- Social AI companions make users “feel heard” which can reduce loneliness [56–58].
- Feelings of love and passion are also extended to AI companions [59, 60].
- Users see their AI friendships as being long-term and similar to human-to-human friendships, especially in terms of affective and social value [61].
- Chatbots induce a sense of relationship in users and can foster self-disclosure, meeting users’ communication and emotional support needs [62, 63].
- Feelings of bonding with AI companions fade away with time when these are perceived as having low credibility or when users have privacy concerns [64].

## Endnotes

1 Note that DDs could in principle be created using a range of AI technologies; however, we will be focusing on LLM-based DDs in this paper,

2 This broadly corresponds to what has been called function-specific and function-broad digital duplicates: see Danaher and Nyholm [2].

3 This brings forth the intuitive nature of privacy as contextual integrity [47].

4 Of course, that is not necessarily the case, as there are also people with malicious intent that want to instrumentalize others by making them believe that they care.
